# Vaccination Against Tuberculosis: Revamping BCG by Molecular Genetics Guided by Immunology

**DOI:** 10.3389/fimmu.2020.00316

**Published:** 2020-02-27

**Authors:** Stefan H. E. Kaufmann

**Affiliations:** ^1^Max Planck Institute for Infection Biology, Berlin, Germany; ^2^Hagler Institute for Advanced Study, Texas A&M University, College Station, TX, United States

**Keywords:** tuberculosis, vaccine, Bacille Calmette-Guérin, subunit, biomarker, macrophage, T lymphocyte, clinical trial

## Abstract

Tuberculosis (TB) remains a major health threat. Although a vaccine has been available for almost 100 years termed Bacille Calmette-Guérin (BCG), it is insufficient and better vaccines are urgently needed. This treatise describes first the basic immunology and pathology of TB with an emphasis on the role of T lymphocytes. Better understanding of the immune response to *Mycobacterium tuberculosis (Mtb)* serves as blueprint for rational design of TB vaccines. Then, disease epidemiology and the benefits and failures of BCG vaccination will be presented. Next, types of novel vaccine candidates are being discussed. These include: (i) antigen/adjuvant subunit vaccines; (ii) viral vectored vaccines; and (III) whole cell mycobacterial vaccines which come as live recombinant vaccines or as dead whole cell or multi-component vaccines. Subsequently, the major endpoints of clinical trials as well as administration schemes are being described. Major endpoints for clinical trials are prevention of infection (PoI), prevention of disease (PoD), and prevention of recurrence (PoR). Vaccines can be administered either pre-exposure or post-exposure with *Mtb*. A central part of this treatise is the description of the viable BCG-based vaccine, VPM1002, currently undergoing phase III clinical trial assessment. Finally, new approaches which could facilitate design of refined next generation TB vaccines will be discussed.

*“ Commit to advancing research for basic science*, *public health research and the development of innovative products and approaches*, *…*, *without which ending the tuberculosis epidemic will be impossible*, *including towards delivering*, *as soon as possible*, *new*, *safe*, *effective*, *equitable*, *affordable*, *available vaccines*, *…”* Resolution adopted by General Assembly of the United Nations from the High Level Meeting on the fight against TB, 2018 (1).

## Introduction

**FIGURE 1:**
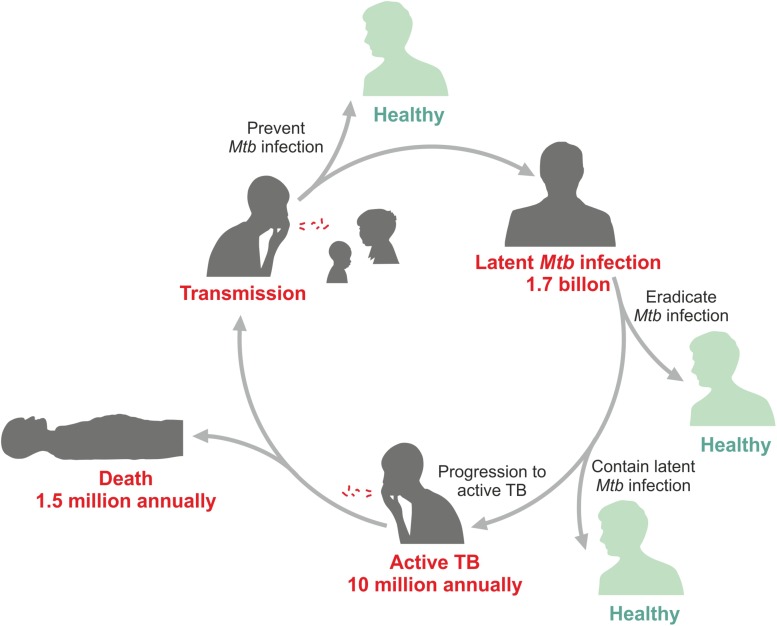
Epidemiologic data for tuberculosis (TB).

The only tuberculosis (TB) vaccine in use until today, Bacille Calmette Guérin (BCG), was introduced in 1921 after intensive research & development (R&D) for more than a decade (2). It was not the first tryout to immunize against TB. The very first attempt was made by Robert Koch who used a subunit-adjuvant formulation (3). Subsequently, several other approaches were tested including killed mycobacterial vaccines and live non-tuberculous mycobacterial strains. Yet, these all failed and the only vaccine with proven safety and efficacy until today remains BCG. In fact, today BCG is the most widely used vaccine, which has been given more than 4 billion times. BCG was developed to protect newborns at high risk of TB (2). This mission has been accomplished at least partially since BCG was proven to protect against severe extra pulmonary, but less against pulmonary TB in infants (4–6). Yet, even today infant TB takes a worrisome toll in TB endemic countries with high coverage of BCG immunization (7–9). Later, BCG was also tested as a vaccine against pulmonary TB in adolescents and adults, but this ambitious target was not reached and no vaccine has ever succeeded in reliably protecting against pulmonary TB, the most prevalent form of the disease, in any age group. A better vaccine is urgently needed since *Mycobacterium tuberculosis* (*Mtb*), the cause of TB, remains on top of the infamous list of deadly infectious agents (10). In 2018, 10 million individuals fell ill with this disease and 1.5 million died (11) (Figure 1). The early 21st century has witnessed increasing R&D efforts for novel TB vaccines (12–19). These include subunit-adjuvant formulations comprising fusion proteins of *Mtb*, viral vectored vaccines expressing one or more antigens of *Mtb*, killed mycobacterial vaccines and viable attenuated mycobacterial vaccines.

## Immunopathology of Tuberculosis

Tuberculosis is a chronic infectious disease caused by the intracellular pathogen *Mtb* (20). This acid-fast bacillus is shielded by a unique lipoid-rich cell wall containing various wax-like substances and glycolipids which contribute to resistance against immune attack. *Mtb* is generally transmitted by aerosols in which it enters alveoli in lower lung lobes. Once the pathogen has been engulfed by alveolar phagocytes, it ends up in a phagosome, where it keeps the local pH neutral (21). Moreover, *Mtb* is capable of egressing into the cytosol (22). These and other mechanisms facilitate resistance of *Mtb* to professional phagocytes including polymorphonuclear neutrophilic granulocytes (in short neutrophils) and mononuclear phagocytes (tissue macrophages and monocytes) (23–27). Resting tissue macrophages generally fail to eliminate *Mtb* and serve as its retreat due to their long lifespan. Blood monocytes are slightly more aggressive but fail to achieve sterile elimination of *Mtb*. Neutrophils are highly aggressive phagocytes with the potential to harm *Mtb*. Due to their short lifespan, they generally will not succeed in completely eliminating *Mtb* and they do not serve as a harbor, in which *Mtb* can persist. Once activated by cytokines, notably Interferon-γ (IFN-γ), mononuclear phagocytes increase their anti-bacterial capacities and pose a more serious threat to *Mtb* although they generally fail to eradicate it completely. The innate immune response mediated by professional phagocytes serves as a first barrier for *Mtb*. Recent evidence suggests that epigenetic changes induced by *Mtb* in professional phagocytes leads to trained immunity. Such trained immunity could play a role in early defense against repeated *Mtb* infections (28, 29). However, thus far compelling evidence for this notion is still incomplete.

In addition, subtypes of dendritic cells (DC) can engulf *Mtb* (30, 31). They likely translocate *Mtb* into the lung parenchyma, where the formation of a granuloma is initiated.

Granuloma formation is strongly regulated by T lymphocytes originally stimulated in the draining lymph nodes to which DC harboring *Mtb* navigate (25, 32). T lymphocytes orchestrate formation of solid granulomas which are primarily composed of macrophages, DCs, and T and B lymphocytes. Within these granulomas *Mtb* is contained and the infected individual remains healthy and develops latent TB infection (LTBI) (24, 33, 34). CD4 T cells have been proven to be central to acquired resistance against and containment of *Mtb* (19, 25). According to the cytokines, these CD4 T cells secrete, they can be categorized into TH1, TH2, and TH17 cells. TH1 cells are preferentially stimulated during *Mtb* infection and are of major importance for defense. They produce cytokines such as IFN-γ, interleukin-2 (IL-2) and tumor necrosis-α (TNF-α). TH2 cells are only weakly induced. They are often considered harmful in TB since they induce inappropriate effector mechanisms. Their major cytokines are IL-4, IL-5, IL-10, and IL-13. However, evidence has been provided that TH2 cytokines, at least in part, can contribute to tissue healing. TH17 cells induce rapid proinflammatory responses by secreting IL-17. They are stimulated during *Mtb* infection and evidence has been published that they participate in protection against TB, notably at early stages of infection. The role of CD8 T cells in protection and containment – although less profound – is also widely accepted. CD8 T cells often produce cytokines of TH1 type and in addition express cytolytic activity (19, 25, 26). Contribution of cytolytic mechanisms to killing of *Mtb* has been demonstrated (35). The role of other lymphoid cells including innate lymphoid cells (iLC), NK T cells, mucosa associated immune T cells (MAIT), γδ T lymphocytes, and B lymphocytes is a matter of ongoing discussion (32, 36–45). B lymphocytes could participate in immunity against TB via two mechanisms: First, as regulatory B lymphocytes and second as antibody producing plasma cells. Evidence for regulatory B lymphocytes in immunity against TB is scarce (46, 47). A role for distinct antibody isotypes in defense against TB has been provided (36, 42, 45). Perhaps these antibodies modulate professional phagocytes through their binding to distinct Fc receptors. Convincing evidence has been generated that γδ T cells contribute to early immune defense by secreting IL-17 (38). The iLC can be categorized into iLC-1, iLC-2 and iLC-3 according to their cytokine secretion pattern (40). Cytokines produced by iLC-1 are of TH1 type, iLC-2 cytokines are of TH2 and iLC-3 cytokines are of TH17 type. The iLC-1 and iLC-3 probably contribute to resistance to *Mtb* and the iLC-2 to healing of lesions (37). During chronic infection, canonical CD4 and CD8 T lymphocytes develop into memory T cells which can be grouped into effector memory T cells (T_EM_), central memory T cells (T_CM_), and tissue resident memory T cells (T_RM_) (48). Although the role of the different memory T cells in protection against *Mtb* is incompletely understood, evidence for a particular role of T_RM_ and T_CM_ in protection against *Mtb* has been provided (49, 50). It is likely that different types of memory T cells participate in protective immunity at different stages of infection.

During LTBI, *Mtb* is contained in solid granuolomas (24, 33, 51). LTBI transforms into active TB disease when granulomas become necrotic and then caseous. This happens in about 5% of individuals with LTBI within the first 2 years and in another 5% at later time points. Thus, only ca. 10% of the 1.7 billion individuals with LTBI develop active TB disease (52). Progression to active disease is due to weakening of the immune response via several incompletely understood mechanisms. It is likely that myeloid-derived suppressor cells and regulatory T lymphocytes participate in dampening of protective immunity (53, 54). These cells produce inhibitory cytokines including IL-4, IL-10, and transforming growth factor-β (TGF-β). Moreover, excessive checkpoint control through inhibitory surface molecules including PD-1/PDL-1 and CTLA-4/B7 co-receptor interactions is likely involved (55, 56).

Notably, progression to active TB from LTBI must be viewed as a continuum rather than a discrete step from one to another stage (33, 57, 58). *Mtb* is transmitted from a TB patient to a healthy individual in a metabolically active and replicative stage. Hence, the host first encounters highly active *Mtb* (24). During LTBI, *Mtb* changes from a metabolically active and replicative stage into a dormant stage in which its activities are markedly downregulated. Once progression to active TB has ensued, *Mtb* wakes up and becomes active again.

At the early stage of infection, it is possible that *Mtb* is rapidly eradicated before stable LTBI develops, but the proportion of individuals who become transiently infected, sometimes accompanied by a short episode of clinical symptoms remains unclear (51, 57, 59). Recent evidence suggests LTBI is succeeded by incipient TB, in which the host remains healthy, but becomes alerted and *Mtb* regains its metabolic and replicative activities (59–62). Subsequently, subclinical TB evolves in which first signs of pathology occur although clinically the patient appears healthy. Signs of host vigilance and pathology can be detected by sensitive gene expression and metabolic profiling (26, 60–62). Given that most, if not all, cases of subclinical TB progress to active TB disease which can be clinically diagnosed, it is possible to predict disease by sensitive profiling by means of transcriptomics and metabolomics (60–63). Note that the different stages are not discrete and that in a single patient areas reflecting LTBI (solid granulomas containing dormant *Mtb*), incipient TB (solid to necrotic granulomas in which *Mtb* regains its metabolic and replicative activity), subclinical TB (further increase in pathology due to transition of some solid granulomas to necrotic ones and eventually first signs of caseation) and active TB (all three forms of granulomas present with a preponderance toward caseation and cavitation) can coexist. Accordingly, different stages of granulomas ranging from solid form to caseation and cavitation coexist, as well (58). Obviously, the coexistence of different pathologies and different *Mtb* activities render TB immunopathology highly complex.

**Box 1. Major vaccine candidates in clinical trials.**

Different types of TB vaccines have entered the clinical trial pipeline. These are: viral vectored protein antigens of *Mtb*, fusion protein antigens of *Mtb* in adjuvants, killed whole mycobacterial cell vaccines, and recombinant viable mycobacterial vaccines. The viral vectored and the adjuvanted protein vaccines are subunit vaccines, which are generally considered to boost a prime with BCG. The viable TB vaccines are considered for BCG replacement or for boosting previous BCG prime. Killed whole cell vaccines are sometimes considered for booster vaccination and more often for TB therapy in adjunct to chemotherapy.

• **Viral vectored** vaccines include MVA85A, a modified vaccinia Ankara (MVA) vaccine expressing antigen Ag85A of *Mtb.* First phase IIb efficacy trials with this vaccine in neonates and in adults failed to provide protection (102, 103). More recently, the vaccine has been tested for safety and immunogenicity after aerosol application (104, 105). Other viral vectored vaccines include replication deficient adenovirus vectors expressing antigen Ag85A and a replication deficient H1N1 influenza vector expressing antigen Ag85A and ESAT-6. Novel prime boost schedules are also being tested including adenovirus vectors for prime and MVA vector for boost expressing antigen Ag85A.
Major viral vectored candidates undergoing clinical testing are:
**Ad5Ag85A** (phase I), a replication-deficient adenovirus (Ad) 5 vector expressing Antigen 85A (106, 107).
**ChAdOx1.85A + MVA85A** (phase I), a prime/boost regimen comprising prime with a chimpanzee Adenovirus (ChAd) expressing Antigen 85A (ChAdOx1.85A) followed by a boost with modified Vaccinia Ankara virus (MVA) expressing Antigen 85A (108).
**TB-FLU-04L** (phase IIa), a replication-deficient H1N1 influenza virus strain expressing Antigen 85A and ESAT-6 (109).
• Protein adjuvant formulations undergoing clinical testing include:
**Hybrid 1** (**H1**, phase I completed) comprising either **IC31** or **CAF01** as adjuvant and a fusion protein of Antigen 85B and ESAT-6 as antigen (110, 111).
**H4** (phase II completed) and **H56** (phase IIb) formulated in **IC31** as adjuvant and fusion proteins of Antigen 85B and TB10.4 (H4) or Antigen 85B, ESAT-6 and Rv2660c (H56) (73, 112–114).
**ID93** (phase IIa) composed of **GLA-SE** as adjuvant and a fusion protein of 4 antigens, namely Rv2608, Rv3619, Rv3620 and Rv1813 (115, 116).
**M72** (phase IIb completed) composed of **AS01_E_** as adjuvant and a fusion protein of 2 antigens, Rv1196, and Rv0125. M72 has completed a phase IIb trial revealing its partial protective efficacy (for further details see text) (65, 66, 117).
• Compositions of adjuvants:
**IC31**, cationic peptides plus TLR-9 agonist;
**CAF01**, cationic liposome vehicle plus immunomodulatory glycolipid;
**GLA-SE**, Squalen oil-in-water emulsion plus TLR-4 agonist;
**AS01_E_**, liposomes with monophosphoryl lipid A plus saponin QS21.
• Viable vaccines undergoing clinical testing are:
**MTBVAC** (phase IIa completed), a genetically attenuated *Mtb* vaccine (118, 119).
**VPM1002** (several phase III trials), a **rBCG** vaccine (for further details see text) (84, 85).
• Killed whole cell vaccines include:
**DAR-901** (killed *M. obuense*) which had already completed a phase III trial under a different name (120–123) and is now under re-evaluation (phase I trial completed) (124).
**MIP** (phase III) based on killed *M. indicus pranii* organisms (125–127).
***M. vaccae*** (phase III) based on killed *M. vaccae* (128–132).
**RUTI** (phase IIa) a purified killed vaccine of *Mtb* fragments (133–135).
• Therapeutic vaccines: The above vaccine trials assess outcome of preventive vaccination. Several candidates are also tested as therapeutic vaccines either for TB treatment in adjunct to canonical chemotherapy or for PoR of TB patients who were cured from TB by canonical chemotherapy but may undergo recurrence (136).
Therapeutic vaccines in clinical trials include:
**H56:IC31** (phase I), a subunit protein formulation;
**ID93:GLA-SE** (phase I), a subunit protein formulation;
**RUTI** (phase IIa), a purified killed vaccine of *Mtb* fragments;
**TB-FLU-04L** (phase IIa), a viral vectored vaccine;
**MIP** (phase III completed), a killed *M. indicus pranii* preparation;
***M. vaccae*** (phase III completed), a killed *M. vaccae* preparation;
**VPM1002** (phase III), a live rBCG vaccine.

## Current Status of Tuberculosis Epidemiology and the Tuberculosis Vaccine Pipeline

According to the latest TB report of the World Health Organization (WHO), 10 million individuals developed active TB disease and 1.5 million died of TB in 2018 (11). Globally 1.7 million individuals are *Mtb* infected (LTBI, incipient TB, subclinical TB) (52). Thus, the goal of the WHO to eliminate TB over the next decades requires much better intervention measures and notably a highly efficacious vaccine (10). BCG fails to protect against pulmonary TB, which is not only the most prevalent form of disease but also the major source of transmission. This has led to several attempts to design novel vaccination regimens (18). Numerous vaccine candidates have entered clinical trials and first promising results have been obtained (see below). Current vaccine candidates undergoing clinical testing are viral vectored vaccines expressing a few *Mtb* antigens, adjuvanted subunit vaccines typically comprising fusion proteins representing two to four *Mtb* antigens, killed whole cell vaccines and viable whole cell vaccines. Further details can be found in Box 1. The vaccine candidates are tested in different clinical situations. These are:

(i) Prevention of Infection (PoI): This clinical endpoint can be applied for pre-exposure vaccination, i.e. vaccination of individuals who have not yet encountered *Mtb*. The most important target group for PoI are neonates. The WHO has prioritized a vaccine to lower the risk of *Mtb* infection (11).
(ii) Prevention of Disease (PoD): It is obvious that PoI will result in PoD. The major target population for PoD, however, are individuals with LTBI. Cutting the risk of TB disease in individuals with LTBI has also been prioritized by the WHO (11).
(iii) Prevention of Recurrence (PoR): In high endemic areas, ca. 10% of TB patients who had been cured by canonical drug treatment undergo recurrence, either due to reinfection or relapse (64).
(iv) Therapeutic Vaccination in Adjunct to Canonical Drug Treatment: Such a vaccination regimen gains increasing importance for patients with multi or extensively drug-resistant TB (MDR / XDR-TB) (16). An estimated half million of active TB patients suffer from MDR-TB and 50,000 to 100,000 individuals from XDR-TB (1). Vaccines for PoR are sometimes considered as therapeutic vaccines, as well.

This review will focus on vaccines that prevent active TB either through PoI, PoD, or PoR.

## Prevention of Disease by the Subunit Vaccine M72 in a Phase IIb Clinical Trial

The M72 vaccine candidate developed by GlaxoSmithKline has successfully completed a phase IIb clinical trial (65, 66). Participants of this study were HIV^–^ adults with LTBI who had been immunized with BCG as infants. Hence, the study was a post-exposure booster immunization with a subunit vaccine with PoD as clinical endpoint. The clinical endpoint was determined after 2 years of follow-up as pulmonary TB in absence of HIV infection (66). The study revealed ca. 50% protection over placebo control. The follow-up study confirmed the efficacy after the third year (65). This is the first vaccine trial to provide evidence for PoD in human TB. A positive control with BCG was not included in this study. It is hoped that global gene expression profiling and immunologic data will provide information about potential mechanisms underlying PoD induced by this vaccine. The vaccine comprises two TB antigens formulated in a potent adjuvant, AS_01E_ (see Box 1). This adjuvant had been developed as part of the adjuvant system (AS) series and is also used in the shingles vaccines, Shingrix, and the malaria vaccine, Mosquirix (67). Availability of AS_01E_ is limited and production cost is high. It has to be seen whether and how these limitations affect supply of this vaccine for broad-scale immunization programs. Satisfactory supply of vaccines for poverty-related diseases including TB and malaria strongly depends on an affordable price (68).

## Promising Prevention of Disease Data in Non-Human Primates (NHP) by a Viral Vectored Tuberculosis Vaccine Candidate

Cytomegalovirus (CMV) based vaccines have been studied in a number of infectious diseases (69). Notably, in a simian immunodeficiency virus (SIV) model of rhesus macaques, CMV vectored vaccines expressing SIV antigens have shown profound protection mediated by CD4 and CD8 T lymphocytes (69). These T cells have been characterized as effector T_EM_ cells and transitional effector memory T cells. Based on these findings, a TB vaccine candidate was designed which is based on a CMV vector expressing 6 or 9 *Mtb* antigens (70). This vector has been tested for PoD in rhesus macaques and was shown to induce profound protection against TB disease (70). Importantly, in a proportion of animals, evidence for sterile eradication of *Mtb* by this CMV-vectored TB vaccine was obtained. As expected, the vaccine induced profound CD4 and CD8 T cell responses as well as marked IFN-γ and TNF secretion. In contrast, antibody responses were not induced significantly. The protective CD8 T cell population was not only restricted by MHC I, but also by MHC-E or MHC II. BCG administered intradermally also induced protection, albeit weaker. Intriguingly, prime with BCG and boost with the CMV-based TB vaccine reverted the strong protective effect of the CMV vaccine to levels of protection induced by BCG. Gene expression profiling of vaccinated animals indicated a role for neutrophils in protection induced by the CMV vectored TB vaccine. In conclusion, despite certain disadvantages of CMV-vectored vaccines in general, the CMV-based TB vaccine represents a promising candidate which deserves further investigation. Obviously, the nullifying effect of BCG prime on protective efficacy induced by the CMV TB vaccine boost needs particular attention. Neonatal BCG immunization is done routinely in high TB endemic areas as part of the expanded program of immunization (EPI) recommended by WHO. Hence a new vaccine that provides no added value for BCG-immunized individuals will face major issues before it can be further developed. Similarly, a recent study revealed that in NHP boosting BCG with M72 or H56 (see Box 1) vaccines failed to enhance protection induced by BCG (71).

## Recent Findings With the Canonical Bacille Calmette-GuÉRin Vaccine

Two recent studies on BCG immunization have revealed marked impact of the vaccination regimen (72, 73). In the first study, NHP were immunized with BCG intravenously (72). Earlier research in the 1970s had already provided compelling evidence that intravenous immunization with live BCG induces superior protection against TB as compared to other routes of administration in NHP with evidence for sterile eradication of *Mtb* (74, 75). Thus, in one study 3/3 animals were markedly protected against TB as measured by hematogenous spread, lymphadenopathy and lung involvement (74). On the other hand, profound splenomegaly was reported after intravenous administration of live BCG. Probably this significant adverse event was the major reason that such studies were not followed up. Only very recently this approach was investigated in greater depth. It was shown that intravenous immunization of NHP with BCG induced more profound protection than intradermal or aerogenic vaccination (72). Indeed, from a proportion of animals receiving BCG by the intravenous route no *Mtb* could be recovered. This study also included a series of highly sophisticated immunologic and pathologic analyses. It was found that antigen responses of CD4 and CD8 T lymphocytes were induced substantially by intravenous immunization prior to *Mtb* challenge, whereas γ/δ T cells and MAIT cells were, similarly, activated as in groups receiving other routes of immunization. The T cell response was mostly of TH1 type with some contribution of TH17 type. On the negative side, splenomegaly was observed after intravenous immunization with a ca. twofold enlargement of spleens compared to controls. However, splenomegaly was transient and 6 months after BCG immunization no differences were observed in spleen size across the different experimental groups including intravenous administration. Six months after immunization, animals were challenged with a low dose of *Mtb*. Positron emission tomography – computed tomography (PET/CT) scans revealed fewer granulomas in the intravenously immunized animals compared to controls. These findings provide proof of concept that BCG immunization can induce profound, in some cases sterile, protection in NHP. It needs to be seen how far the splenomegaly observed will be prohibitive for clinical studies in humans.

The second recent study tested the outcome of BCG booster vaccination in *Mtb* unexposed adults (73). Booster vaccination with BCG had been performed previously although generally it was not endorsed because of the potential risk of adverse events. This assumption was largely based on anecdotal reports describing occasional adverse events after repeated BCG immunization in individuals with LTBI and frequent severe events in TB patients. Principally, BCG revaccination in *Mtb* uninfected individuals does not cause major side effects and in the recent formal clinical trial, BCG revaccination of *Mtb* unexposed individuals demonstrated partial prevention of stable *Mtb* infection (73). More precisely, exposure was determined indirectly via an IFN-γ release assay (IGRA) which determines IFN-γ secretion by canonical T cells after *in vitro* restimulation with *Mtb* specific antigens (76–78). This assay is mostly based on CD4 T cell responses with some contribution of CD8 T cells. Whilst initial IGRA conversion did not differ between BCG immunized and untreated study participants, sustained IGRA conversion was significantly reduced by ca. 45% in BCG immunized study participants over controls (73). These findings can be interpreted to mean that stable *Mtb* infection is prevented by BCG revaccination although in fact it is based on reduced T cell responses as measured by IGRA. It remains to be established more precisely whether prevention of sustained IGRA conversion directly translates into long-term PoI and consequently PoD. Previous observational studies had evaluated PoD by BCG revaccination based on epidemiologic data. Generally, they did not find significant differences between controls and BCG revaccinated individuals (79–81).

These two studies provide strong evidence that the outcome of BCG vaccination is markedly influenced by the kind of administration, notably route of immunization (intravenous) and type of vaccine schedule (pre-exposure revaccination). In conclusion, the BCG vaccine still provides room for improvement.

## VPM1002

One of the most advanced TB vaccines, VPM1002, was improved by genetic modification (82). VPM1002 is a recombinant BCG (rBCG) which expresses listeriolysin from *Listeria monocytogenes* and is devoid of urease C (83). Development of this vaccine had started in the 1990s with the aim to improve BCG by endowing it with the capacity to stimulate a broader, more efficacious T cell response.

VPM1002 has successfully completed phase I and phase IIa clinical trials proving its safety and immunogenicity in adults and neonates (84, 85). A phase II clinical trial in HIV exposed and unexposed neonates has been completed and awaits unblinding (NCT 02391415). A phase III clinical trial in HIV exposed and unexposed neonates is being prepared and expected to start in 2020. This trial has been designed as pre-exposure BCG replacement for infants with PoI as clinical endpoint. In this clinical trial, termed **priMe**, neonates will be immunized with VPM1002 or BCG as comparator at several sites in Sub-Saharan Africa. A phase III clinical trial with VPM1002 assessing PoR is currently ongoing in India (NCT 03152903). For this trial, patients with TB who had been cured by drug treatment are being recruited. An estimated 10 % of these individuals will develop active TB disease due to reinfection or relapse within 1 year after completion of drug treatment. The clinical trial therefore will reveal whether vaccination with VPM1002 given 3 months after completion of drug treatment can prevent recurrence. A phase III household contact trial has been launched in July 2019 by the Indian Council for Medical Research (ICMR), in which VPM1002 and another vaccine candidate (MIP, see Box 1) will be assessed for PoD in household contacts of patients with active pulmonary TB disease. In addition, VPM1002 is also being assessed as therapeutic agent against non-muscle invasive bladder cancer as substitute for BCG (NCT 02371447). The canonical TB vaccine BCG is the preferred immunomodulatory medicine for treatment of bladder cancer and the current clinical trial assesses whether VPM1002 is safer than and at least equally efficient as BCG against recurrence of bladder cancer. In conclusion, a century after the introduction of the original vaccine BCG, there is hope for a revival of an improved BCG-based TB vaccine. A rationally revamped BCG could contribute to the solution of the TB crisis.

## How Does the Intracellular Behavior Differ Between VPM1002, Bacille Calmette-Guérin, and *Mtb*?

**FIGURE 2:**
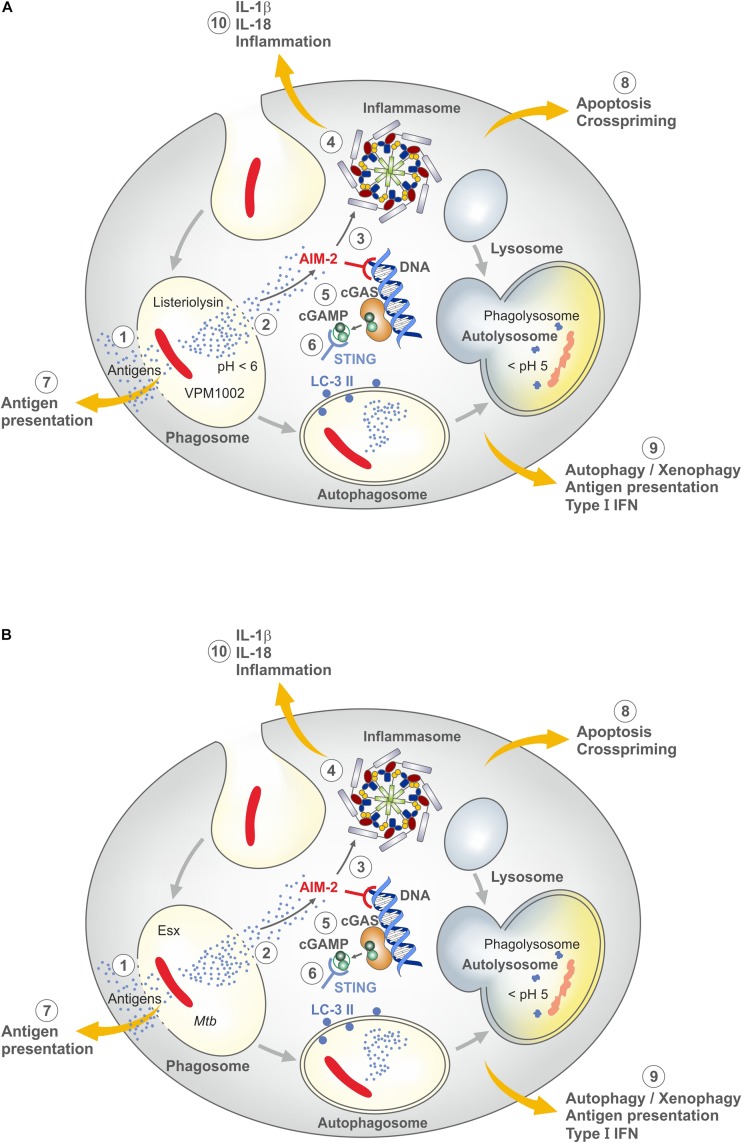
Major mechanisms underlying induction of the host immune response by VPM1002 and *M. tuberculosis* (*Mtb*) (for further details see text). **(A)** VPM1002. VPM1002 (rBCGΔureC::Hly) expresses listeriolysin and lacks urease C activity. Following phagocytosis, VPM1002 ends up in a phagosome. Principally phagosomes become acidic after uptake of particles, but BCG and *Mtb* actively keep the phagosomal pH neutral. Due to the absence of ureaseC in VPM1002, acidification takes place. This facilitates perturbation of the phagosomal membrane by biologically active listeriolysin. (1) Membrane perturbation allows egress of antigens into the cytosol for processing through the MHC class I pathway. (2) Perturbation can lead to apoptosis. (3) Double-strand DNA released into the cytosol is sensed by absence in melanoma 2 (AIM2). (4) AIM2 activates the inflammasome to generate IL-1β and IL-18. (5) Cyclic GMP-AMP synthase (cGAS) is formed which is then transformed into cyclic guanosine monophosphate-adenosine monophosphate (cGAMP). (6) The latter molecule is sensed by stimulator of IFN genes (STING) which induces autophagy and type I IFN responses. (7) Antigen egress into the cytosol allows stimulation of CD8 T cells in addition to CD4 T cells. (8) Apoptosis promotes crosspriming. (9) Autophagy accelerates elimination of VPM1002 and improves antigen presentation and T cell stimulation. (10) IL-1β and IL-18 induce an inflammatory response. Through these mechanisms, VPM1002 induces an immune response with more depth and breadth than parental BCG **(B)** *Mtb*. The genome of *Mtb* comprises the region of difference 1 (RD-1) which encodes numerous virulence factors which are absent in BCG. Notably genes for Esx dependent mechanisms cause perturbation of phagosomal membranes, very similar to VPM1002. For further details see **(A)**. Because the RD-1 encoded gene products are not degraded after their egress into the cytosol, pathologic consequences prevail. Moreover, RD-1 encoded gene products are not controlled by pH. Hence, inbuilt safety mechanisms of VPM1002 are absent from *Mtb* (see also Figure 3).

Both BCG and *Mtb* reside in phagosomes, which are arrested at an early stage by neutralization of the phagosomal pH to prevent its acidification (21). Consequently, phagolysosome fusion is diminished. Yet, BCG is degraded in the phagosome whereas *Mtb* survives in phagocytes for prolonged periods of time. Only recently, virulence mechanisms of *Mtb* absent from BCG have been elucidated. Although several sub-strains of BCG exist, it is now clear that the critical step which occurred during attenuation of the parental *Mycobacterium bovis* strain was the loss of the region of difference (RD) 1 which encodes a number of gene products mediated through the ESX/type VII secretion system and capable of perturbating the phagosomal membrane (86). Membrane perturbation by the RD-1 gene products of *Mtb* leads to inflammasome activation, apoptosis and autophagy (Figure 2). The signaling cascades involve nod-like receptor protein 3 (NLRP-3) and absent in melanoma 2 (AIM-2), responsible for IL-1 and IL-18 processing from their respective precursor molecules by the inflammasome as well as STING responsible for autophagy and type I IFN dependent responses (87). STING senses cyclic guanosine monophosphate-adenosine monophosphate (cGAMP) derived from double-stranded DNA of *Mtb* via the enzyme cyclic guanosine monophosphate-adenosine monophosphate synthase (cGAS). All these sequelae are caused by *Mtb* but not or less so by BCG.

**FIGURE 3:**
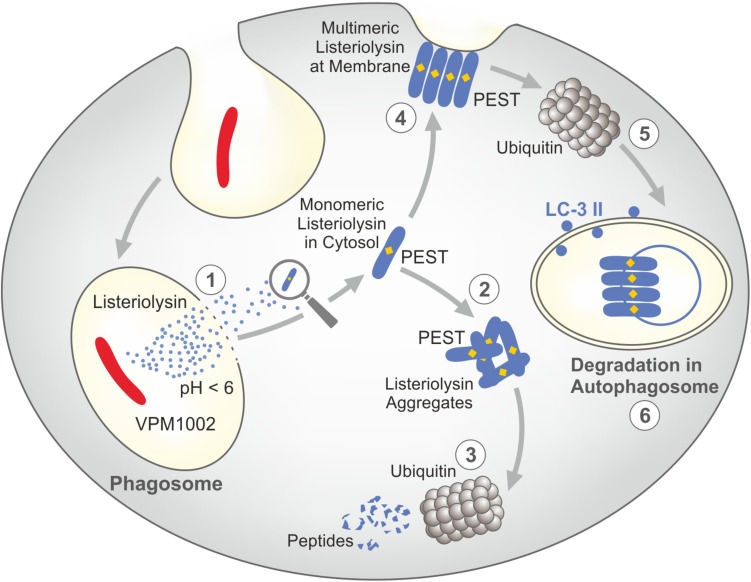
Safety mechanisms of listeriolysin render VPM1002 less virulent than parental BCG. Listeriolysin contains a PEST-like sequence which promotes its degradation. (1) Only at acidic pH, listeriolysin is biologically active and hence perturbates the phagosomal membrane. (2) In the cytosol, monomeric listeriolysin aggregates. (3) Aggregated listeriolysin is degraded by ubiquitin resulting in inactive peptides. (4) Multimeric listeriolysin complexes are formed at the plasma membrane. (5) These complexes are translocated into autophagosomes by ubiquitin. (6) These listeriolysin complexes are inactivated in the phagosome. PEST = Proline (P), Gutalate (E), Serine (S), and Threonine (T). Modified from (88–90).

For the design of VPM1002, BCG was equipped with listeriolysin from *L. monocytogenes* which facilitates perturbation of the phagosomal membrane thereby inducing stronger T cell responses (83). Listeriolysin is a thiol-activated perforin, which perforates cholesterol containing membranes at an acidic pH (88–90). This pH restriction generally prevents listeriolysin activity in the extracellular milieu with neutral pH, e.g. blood and interstitial space. It is however, achieved during natural infection of phagocytes with *L. monocytogenes* which allows secretion of biologically active listeriolysin. Because BCG neutralizes the phagosomal compartment, acidification is not achieved. For this reason, the urease C encoding gene was deleted in VPM1002 (83). This enzyme is responsible for ammonia production and thereby participates in neutralization of the phagosome where BCG resides (21). Accordingly, VPM1002 lacking urease C favors phagosomal acidification and thereby secretion of biologically active listeriolysin (Figure 2). Once listeriolysin has reached the cytosol, it is rapidly degraded. This is due to the amino acid sequence proline-glutamate-serine-threonine (PEST) in the listeriolysin amino acid sequence which promotes its ubiquitination (Figure 3) (88–90). This represents an inbuilt safety mechanism, which restricts listeriolysin-activity to the perturbation of the membrane of the phagosome, where VPM1002 resides and prevents further potentially detrimental effects on cell membranes. The RD-1 encoded machinery of *Mtb* is not endowed with such a safety mechanism.

Similar to the RD-1 machinery in *Mtb*, listeriolysin-mediated perturbation of the phagosomal membrane by VPM1002 results in inflammasome activation through AIM-2 (91). Hence, IL-1 and IL-18 are processed from their respective precursors. These proinflammatory cytokines create a milieu favorable for activation of TH1 and TH17 cells. Listeriolysin also facilitates autophagy via AIM-2 and STING by promoting sensing of double-strand mycobacterial DNA derived from VPM1002 via cGAS and cGAMP after its egress into the cytosol (91). In addition, membrane perturbation by listeriolysin causes apoptosis, which leads to cross priming of T cells (92). Together these mechanisms improve vaccine efficacy of VPM1002 as compared to canonical BCG (82, 93). Moreover, VPM1002 was shown to be safer than BCG in preclinical studies (83). In experimental models improved stimulation of both CD4 and CD8 T cells has been demonstrated (92) as well as more profound activation of TH17 cells in addition to TH1 cells (94). In addition, central memory T cells were more strongly stimulated by VPM1002 as compared to canonical BCG (50). Finally, VPM1002 was shown to stimulate higher serum levels of specific antibodies both in animal models and in human (50, 84, 85). In conclusion, VPM1002 stimulates an immune response of more depth and breadth and at the same time expresses lower virulence as compared to BCG (82).

## Learning From Individuals Resistant to Stable *Mtb* Infection and Those Capable of Eradicating *Mtb* After Stable Infection

**FIGURE 4:**
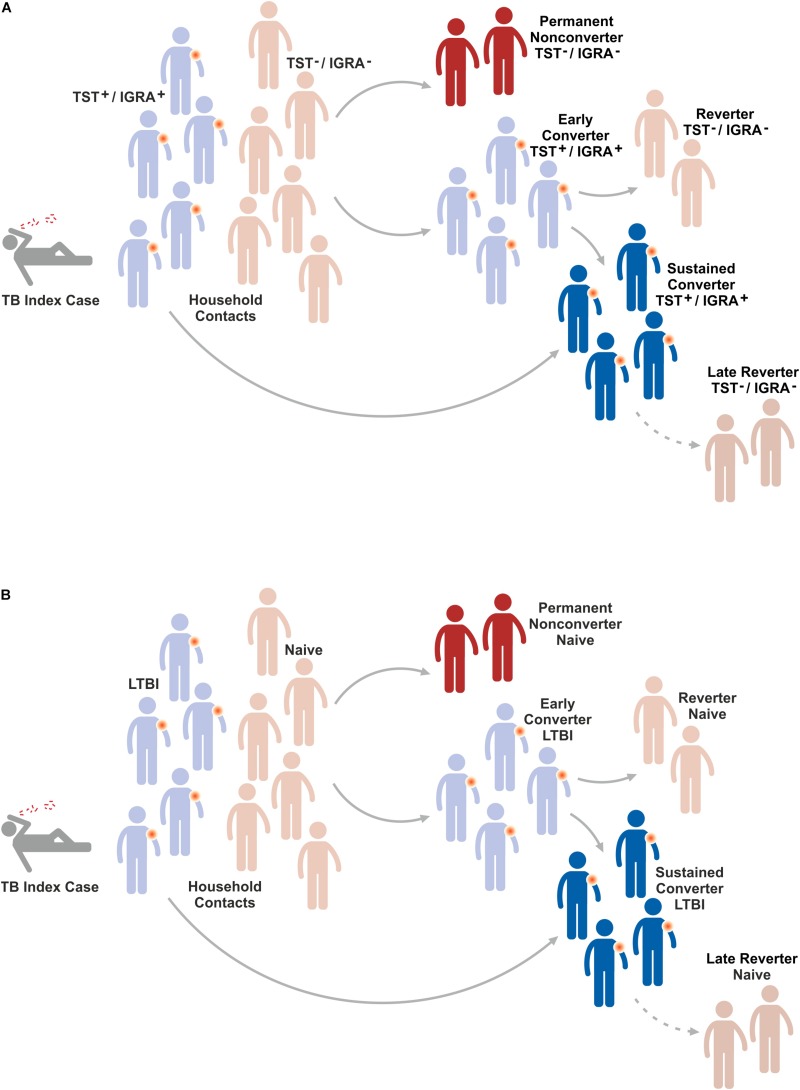
Fate of household contacts of a TB index case. Household contacts of a TB index case are either already latently TB infected (LTBI) or do not show evidence for immunity against *Mtb* infection. After sustained contact with a TB index case, the majority of naïve individuals will rapidly convert to LTBI because they mount an immune response against *Mtb* infection. Most of these early converters will remain LTBI and hence become sustained converters. A small proportion of early converters reverts to naïve, i.e. devoid of a measurable immune response to *Mtb* infection. Some naïve individuals will remain permanent non-converters, i.e. they do not change their status of absent immunity indicating absence of *Mtb* infection. Finally, some individuals with LTBI will revert to naïve, i.e. they lose their detectable immune response to *Mtb* indicating elimination of *Mtb*. The mechanisms underlying these conversions/reversions remain elusive. **(A)** Indicates response in TST/IGRA and **(B)** depicts resulting conclusions on conversion/reversion (for further details see text).

Individuals with LTBI are generally identified by tuberculin skin test (TST) or IGRA (Figure 4) (76–78). Accordingly, identification of the 1.7 billion individuals on this globe with LTBI is based on measurements of T cell responses against *Mtb* antigens. These antigens are relatively undefined mixtures of *Mtb* proteins (purified protein derivative, PPD) in the case of TST and well defined *Mtb* proteins and/or peptides in the case of IGRA.

Several lines of evidence suggest that a distinct population of individuals remain *Mtb* uninfected despite their close and prolonged contact with patients with active pulmonary TB who continuously expel *Mtb* (95–98). This notion is based on the finding that such individuals do not convert when tested by TST or IGRA. Assuming that the lack of the canonical immune response determined by TST and/or IGRA reflects absence of *Mtb* infection, the following scenario arises (Figure 3): Initially, household contacts of a patient with active pulmonary TB fall into two groups; those who are already LTBI because of previous contact and hence are TST^+^/IGRA^+^, and naïve individuals who are TST^–^/IGRA^–^. Due to the intensive contact, most of the naïve individuals will convert to TST^+^/IGRA^+^ and most of them remain TST^+^/IGRA^+^ over longer periods of time, if not lifelong. However, a small group may revert to TST^–^/IGRA^–^ indicating that they are capable of eradicating *Mtb* before they become permanently infected. The recent BCG revaccination trial on PoI (73) described above did not reveal significant differences between BCG-immunized and control groups in early conversion to IGRA^+^. Yet, a 45% reduction in sustained IGRA^+^ (determined at later time points) was observed in the BCG immunized group as compared to controls without BCG immunization. Moreover, observational studies have identified a distinct group of permanent non-converters (TST^–^/IGRA^–^) generally in the order of 20% (95–98).

Obviously, the described effects could also be due to technical reasons and the TST^–^/IGRA^–^ group could be infected with *Mtb* but missed by TST/IGRA because these individuals develop a protective immune response which is not detected by TST and IGRA. Underlying mechanisms could include antibodies, MAIT cells, γδ T cells, NK cells and NKT cells (32, 36–45). Furthermore, it remains unclear whether all TST^+^/IGRA^+^ individuals are indeed *Mtb* infected or whether at least a subgroup has succeeded in eliminating *Mtb* but remains TST^+^/IGRA^+^ because of a strong memory T cell response which persists in absence of *Mtb* antigens. Principally, immunology defines memory as a state of immunity in absence of nominal antigen(s).

Another interesting group may arise years after primary *Mtb* infection. Whilst many individuals with LTBI remain TST^+^/IGRA^+^ livelong, some individuals revert to TST^–^/IGRA^–^. It is likely that in these individuals, reversion from TST^+^/IGRA^+^ to TST^–^/IGRA^–^ reflects sterile eradication of *Mtb*. Yet, it cannot be excluded formally that these individuals remain *Mtb* infected and control infection by unknown immune mechanisms not detected by TST/IGRA such as antibodies and unconventional T cells. In any case, the permanent non-converters and the late reverters are highly interesting study groups which provide the opportunity to gain deeper insights into the mechanisms of protection against *Mtb*. TB vaccines which prevent stable infection with *Mtb* and thereby prevent LTBI and active TB disease would be highly desirable. The specific mechanisms underlying permanent non-conversion and late reversion could be elucidated by determining transcriptomic, metabolomic and immunologic markers and signatures which distinguish permanent non-converters and late reverters from sustained and livelong reverters, respectively (26, 99).

## Outlook and Future

Over the last decade, the TB vaccine pipeline has significantly progressed. First, a number of vaccines is ready for clinical efficacy testing for PoI, PoD, or PoR (see Box 1). This implies that several vaccine candidates have already proven their safety and immunogenicity. Second, several positive signals arose from clinical trials over the last years including proof of concept that a subunit vaccine empowered by a strong adjuvant can partially protect against active TB when given post-exposure to individuals with LTBI (65, 66). Third, BCG revaccination of *Mtb* unexposed individuals has provided indirect evidence for partial prevention of sustained *Mtb* infection (73). Obviously, major issues remain to be solved. These include: First, BCG revaccination outcome was determined by IGRA which measures canonical T-cell immune responses rather than *Mtb* infection *per se* (see above). This raises the question whether BCG indeed prevented *Mtb* infection or whether infection occurred but was controlled by alternative immune mechanisms such as antibodies and/or unconventional T cells. Second, the M72 clinical trial did not include BCG vaccination as a positive control. Third, studies in NHP revealed that different subunit vaccine constructs including M72, H56, and the CMV vectored TB vaccine failed to increase protection when given as booster on BCG prime (70, 71). In sum, there is well justified hope for better vaccines; but it remains difficult to predict when and to which degree TB can be controlled by improved vaccination strategies.

**FIGURE 5:**
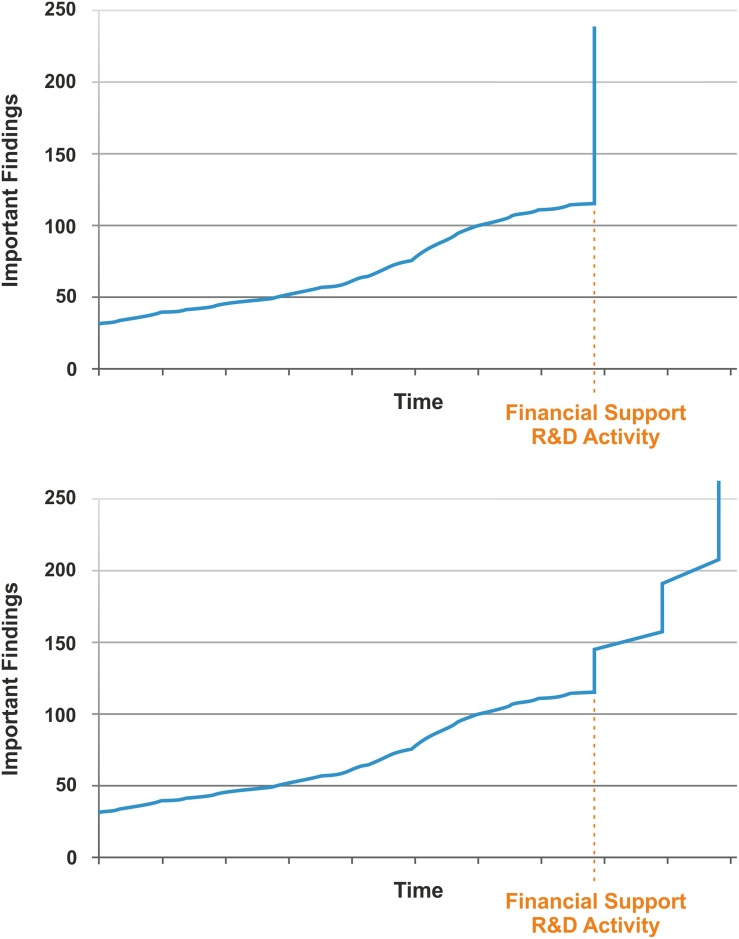
Possible scenarios of TB vaccine development given that adequate financial funding is provided for research & development (R&D). **Upper**, single step event; **Lower**, multistep event.

Nassim Nicholas Taleb is best known for describing the Black Swan Concept which basically includes the notions (100): (i) rare and improbable events do occur more frequently than we assume; (ii) these extreme events can have enormous consequences; (iii) experts generally provide explanations post-hoc which were not plausible ex-ante. This concept was aptly illustrated by the financial stock market crisis in 2008 when numerous stock owners who had gradually accumulated a financial depot went bankrupt through a single event. The most illustrative description for the Black Swan concept is the life of a Thanksgiving Day turkey which is taken care of very well over the first 1000 days by feeding it with most nourishing food. An observer (including the turkey if it could do so) could conclude that the quality of life of this animal increases constantly. Yet, on day 1001, the butcher kills the animal unexpectedly in preparation for Thanksgiving Day. Obviously, this is an extreme event with a major impact on the animal. This scenario can also be turned upside down into a positive direction, i.e. that an unexpected and improbable event turns into something markedly better (an event which would perhaps be better described by the term Pink Swan). With respect to TB vaccine design, continuous funding into R&D (from basic research to preclinical and clinical development) will increase our knowledge about the underlying mechanisms of protection against TB and how this information can be harnessed for TB vaccine design (Figure 5).

For long periods of time research crawls, but every now and then it jumps. By increasing funding, a fertile soil can be prepared for R&D on better vaccines. Maybe this leads to an extreme event (a single jump), resulting in a novel vaccine that fits all purposes. More likely a couple of smaller, yet significant events will occur which ultimately lead to TB vaccines for different purposes. The type of vaccine and the time when it will be ready for clinical licensing remain unclear as expected for a Black/Pink Swan. Yet, increased funding for R&D will favor chances of success. Undoubtedly, this cannot be accomplished free of cost; yet ultimately it will save cost by reducing the enormous expenses caused by the TB endemic. After all, annual cost for treating active TB disease globally has been estimated to be in the range of 2 billion US dollars and the total burden of TB on the global economy in the order of 100 billion US dollars (101).

It is worth to conclude with a citation from the resolution adopted by the General Assembly of the United Nations from the High Level Meeting on the fight against TB in 2018 (1):

*“Commit to mobilize sufficient and sustainable financing*, *with the aim of increasing overall global investments to 2 billion dollars*, *in order to close the estimated 1.3 billion dollar gap in funding annually for tuberculosis research*, *ensuring that all countries contribute appropriately to research and development ….”*

## Author Contributions

SK conceived the idea and wrote the manuscript.

## Conflict of Interest

SK is co-inventor of the TB vaccine, VPM1002 and co-holder of a patent licensed to Vakzine Projekt Management GmbH, Hanover, Germany and sublicensed to Serum Institute of India Pvt. Ltd., Pune, India. The vaccine is currently undergoing clinical trial testing.
